# Evaluation of Stress Distribution in Splinted Crowns on a Hemisected Tooth: A Finite Element Analysis

**DOI:** 10.7759/cureus.112174

**Published:** 2026-07-06

**Authors:** Divyakshita S, Chelva Sharnika M S, Vinoo Subramaniam Ramachandran, Vinaykumar G, Sibi Swamy, Abinaya K

**Affiliations:** 1 Conservative Dentistry and Endodontics, RVS Dental College and Hospital, Coimbatore, IND

**Keywords:** finite element analysis, hemisection, lithium disilicate, mandibular molar, post-endodontic restoration, splinted crowns, stress distribution, von mises stress, zirconia

## Abstract

Objective: The objective of this study was to compare the stress distribution on splinted crowns made of two different materials placed on hemisected teeth using finite element analysis (FEA).

Materials and methods: This study employed FEA to assess the stress distribution on the remaining dentin after placing two types of splinted crowns made of different materials on hemisected mandibular first molar models. Two hemisected mandibular first molar models were used for the study. The root canals of both models were filled with gutta-percha by FEA simulation. The splinted crown was the coronal restoration placed on the hemisected mandibular first molar and its adjacent tooth, the mandibular second molar. In the first model, a lithium disilicate splinted crown was placed, and in the second model, a zirconia splinted crown was placed. Using a three-dimensional finite element model, both crown models were subjected to loading and tested. Static loads of 450 N and 600 N were sequentially simulated on both models in vertical and oblique directions to the occlusal plane, and their maximum von Mises stresses were obtained using FEA.

Results: Under a 450 N load, the maximum stress for the lithium disilicate splinted crown was 60.889 MPa on the adjacent tooth and 49.858 MPa on the hemisected tooth restored with the lithium disilicate splinted crown, whereas the maximum stress for the zirconia splinted crown was 61.421 MPa on the adjacent tooth and 49.034 MPa on the hemisected tooth restored with the zirconia splinted crown. Under a 600 N load, the maximum stress for the lithium disilicate splinted crown was 81.185 MPa on the adjacent tooth and 66.478 MPa on the hemisected tooth restored with the lithium disilicate splinted crown, whereas the maximum stress for the zirconia splinted crown was 81.824 MPa on the adjacent tooth and 65.547 MPa on the hemisected tooth restored with the zirconia splinted crown.

Conclusion: This study concludes that zirconia splinted crowns transferred numerically less stress to the hemisected tooth than lithium disilicate crowns; however, the difference was not statistically significant. The use of splinted crowns in hemisected teeth significantly improves stress distribution and reduces the load on the remaining tooth structure.

## Introduction

Coronal restorations are essential for restoring function and esthetics in teeth that have been structurally compromised due to caries, trauma, or extensive restorative procedures. In severely weakened teeth, conventional single crowns may not always provide adequate support or stability. Splinted crowns offer an effective alternative by joining two or more crowns into a single unit, allowing better distribution of occlusal forces across multiple abutments. This helps reduce stress on individual teeth, improves stability, and enhances long-term prognosis, especially in cases with periodontal weakness, reduced tooth structure, or compromised crown-root ratios. Although highly beneficial, splinted crowns require careful case selection, precise planning, and attention to oral hygiene maintenance because of their more complex design. Overall, they provide a reliable solution for restoring function and durability in challenging clinical situations [1]. Hemisection often refers to the excision of half of a tooth, executed in two stages: tooth sectioning and subsequent extraction of one root. Among these, splinted crowns have become an effective solution for restoring weakened teeth, particularly after procedures such as hemisection [2]. In such cases, splinted crowns, which connect adjacent teeth with a rigid framework, help restore function and prevent further damage. Typically, definitive coronal restorations are placed after complete healing following hemisection surgery to ensure periodontal stability and optimal tissue adaptation. These restorations are intended to restore the function and structural integrity of the remaining tooth while redistributing functional occlusal loads. However, their long-term clinical success depends largely on how effectively stresses are transferred through the restoration to the remaining tooth structure, periodontal ligament (PDL), and supporting alveolar bone. Inadequate stress distribution may lead to localized stress concentrations, increasing the risk of root fracture, restoration failure, or periodontal compromise. Consequently, understanding the biomechanical behavior of different restorative materials under functional loading is essential for selecting the most suitable coronal restoration for hemisected teeth. Finite element analysis (FEA) provides a reliable, noninvasive method for evaluating these stress distribution patterns and predicting the mechanical performance of restorative materials before clinical application.

When a structure is subjected to functional forces, the resulting stress distribution can be effectively analyzed using three-dimensional (3D) FEA. This technique, combined with computer-generated models derived from micro-computed tomography (micro-CT) imaging, allows for highly accurate simulation of complex anatomical and restorative structures. By integrating micro-CT data, precise geometric details of teeth and surrounding tissues can be reconstructed, enabling realistic modeling of material behavior under load. This approach helps in understanding stress concentration areas, predicting failure patterns, and optimizing restorative designs for improved biomechanical performance [3]. FEA is a definitive approach for modeling intricate structures and evaluating their mechanical characteristics. FEA is widely recognized as a noninvasive and reliable method for evaluating biomechanics and understanding how mechanical forces influence biological systems. It enables detailed assessment of stress, strain, and deformation within complex structures under simulated loading conditions. In dentistry and biomedical research, FEA is particularly valuable for analyzing tooth-restoration systems, bone behavior, and implant performance, helping predict mechanical responses without the need for destructive testing [4]. FEA provides precise quantitative data at specific locations within a model through mathematical simulation. In the 3D finite element method, a geometric structure is divided into a finite number of small elements, within which variables of interest are approximated using suitable mathematical functions.

The solid model is then converted into a finite element model (FEM) for computational analysis. In this process, each element is defined and connected at nodal points, forming an interconnected system. The force-displacement relationship, expressed in terms of nodal variables, is used to develop the element stiffness matrix. These individual element matrices are subsequently assembled to form the global stiffness matrix, which governs the overall mechanical behavior of the system [5].

This computational technique breaks down complex geometries into smaller, manageable elements, facilitating the assessment of stress, strain, and deformation patterns in various materials used in dental restorations. Understanding stress distribution is critical for predicting the longevity of splinted crowns on hemisected teeth and ensuring their clinical success.

The purpose of this study was to evaluate the stress distribution of splinted crowns made of different materials on hemisected teeth using FEA. Specifically, the research investigated how different splinting materials and designs affect the mechanical performance of the restoration, with a focus on stress concentrations at critical points such as the remaining tooth structure and PDL. Through this analysis, it may be possible to optimize restorative techniques and materials, thereby improving the outcomes of hemisected tooth restorations.

Several studies have examined the failure behavior of different restorative materials and stress distribution patterns using FEA [6]. However, to the best of our knowledge, no study has directly compared the stress distribution of lithium disilicate and zirconia splinted crowns on hemisected teeth using FEA. Therefore, the present study aimed to compare these two restorative materials.

## Materials and methods

Tooth model and hemisection procedure

The finite element models used in this study were based on the anatomy of human mandibular first and second molars, both of which are frequently involved in clinical restorations. The first molar was used as a model for a healthy tooth, while the second molar was modeled as a hemisected tooth. The 3D geometry of these teeth was derived from high-resolution digital scans of actual molars to ensure accurate anatomic features, including the crown, roots, pulp chamber, and PDL.

In the case of the hemisected tooth, one root and its corresponding portion of the crown were removed to simulate a clinical hemisection procedure. The remaining portion of the tooth (which retained one root) was preserved to maintain the tooth's structure and function, reflecting a typical clinical situation for restoring a compromised molar. The finite element model was developed using the original anatomic dimensions obtained from the 3D scan of the mandibular molar. Consequently, the crown-to-root ratio reflected the natural anatomy of the scanned tooth rather than a predefined value.

Material properties

Each component of the tooth and restoration was assigned material properties consistent with those observed in dental materials and tissues. Enamel was modeled with a Young's modulus of 84 GPa and a Poisson's ratio of 0.30, corresponding to its properties [7]. The underlying dentin was modeled with a Young's modulus of 18.6 GPa and a Poisson's ratio of 0.31, corresponding to its properties [8]. The PDL was modeled with a Young's modulus of 0.1 MPa and a Poisson's ratio of 0.45, corresponding to its properties and reflecting its role in stress distribution and load absorption [9]. Cortical bone was modeled with a Young's modulus of 13.7 GPa and a Poisson's ratio of 0.30, corresponding to its properties and reflecting its role in stress distribution and load absorption [10]. Spongy bone was modeled with a Young's modulus of 1.37 GPa and a Poisson's ratio of 0.30, corresponding to its properties and reflecting its role in stress distribution and load absorption [11].

For the splinted crowns, two types of restorative materials were chosen for the simulations. In Model A, a lithium disilicate splinted crown was used with a Young's modulus of 70 GPa and a Poisson's ratio of 0.25. This material is known for its strength, aesthetic properties, and superior mechanical performance under masticatory loading. In Model B, a zirconia splinted crown was used with a Young's modulus of 210 GPa and a Poisson's ratio of 0.30 [11]. Known for its excellent mechanical properties, zirconia is often chosen for its high fracture toughness and wear resistance, especially in cases of extensive tooth loss.

Model Construction and Finite Element Mesh

**Figure 1:**
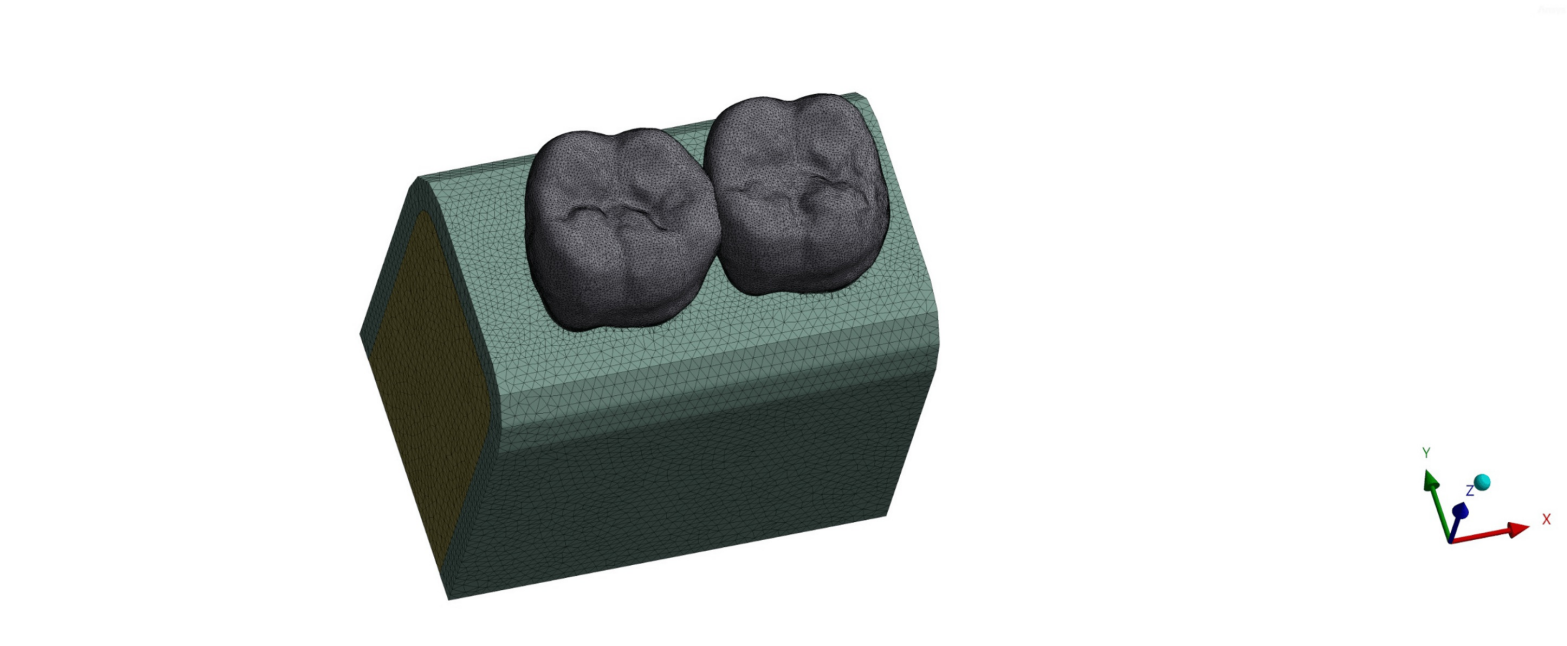
Mesh model: occlusal view Figure was generated by the authors using the SolidWorks software (Dassault Systèmes SolidWorks Corp., Waltham, MA).

**Figure 2:**
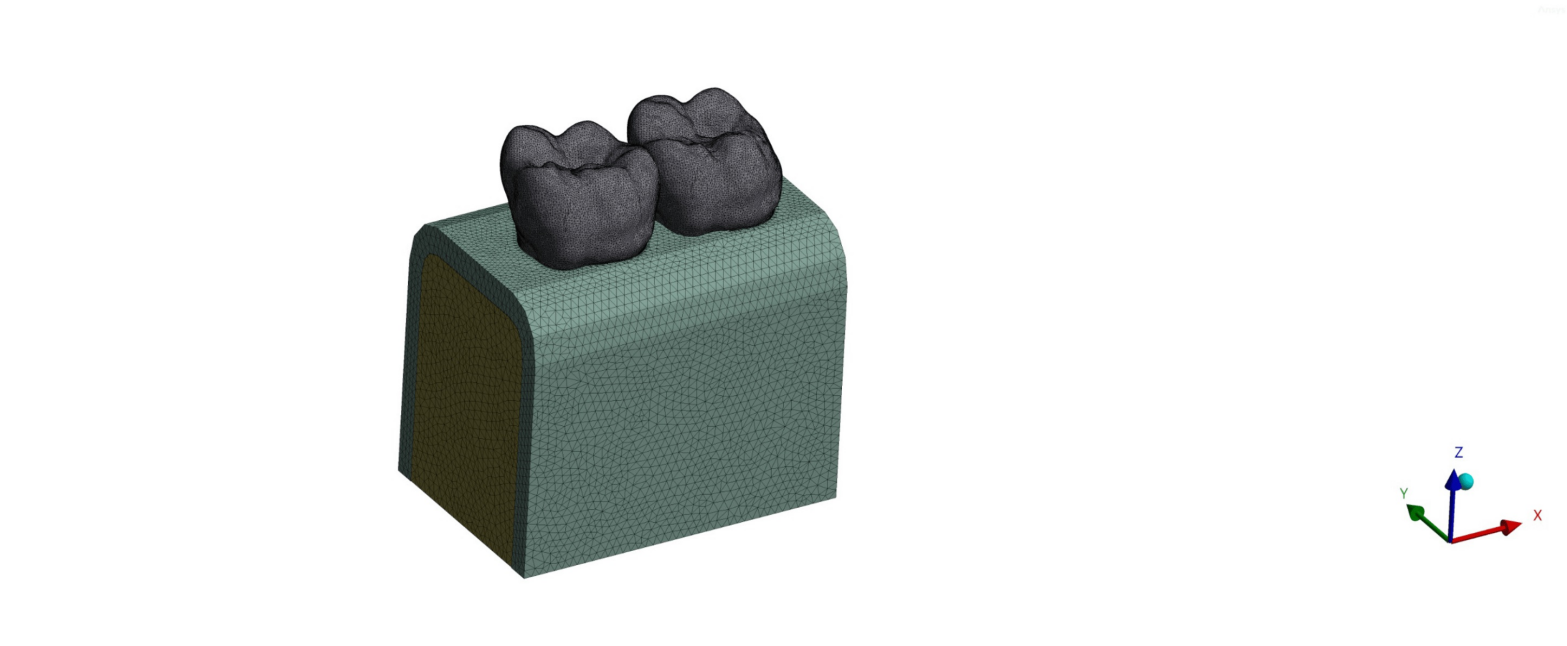
Mesh model: faciolingual view Figure was generated by the authors using the SolidWorks software (Dassault Systèmes SolidWorks Corp., Waltham, MA).

The finite element model was developed using the original anatomic dimensions obtained from the 3D scan of the mandibular molar. Consequently, the crown-to-root ratio reflected the natural anatomy of the scanned tooth rather than a predefined value. Figure 1 shows the occlusal view, and Figure 2 shows the faciolingual view. The shape and dimensions used to model the tooth were obtained from Wheeler's *Dental Anatomy, Physiology, and Occlusion* [12] and *Contemporary Fixed Prosthodontics* [13].

After designing the tooth models, hemisection of the second molar was performed digitally by removing one root and the corresponding portion of the crown, thus simulating a clinical hemisection scenario. All simulations were performed using SolidWorks software (Dassault Systèmes SolidWorks Corporation, Waltham, MA). All simulated models were transferred to ANSYS (Ansys, Inc, Pennsylvania, USA), an FEA software program.

The models were discretized using a finite element mesh of tetrahedral elements. To improve accuracy, a mesh refinement strategy was applied in regions expected to have higher stress concentrations, such as the pulp chamber, dentin-enamel junction, and the PDL interface. The mesh included approximately 1,098,803 elements and 1,684,657 nodes for each tooth model, ensuring that the results accurately captured the mechanical behavior of the tooth and restoration under loading conditions.

Loading and boundary parameters

The loading methodology used in this investigation adhered to the methods established in prior research by Imanishi et al. [14] and Nakamura et al. [15]. Static loads of 450 N and 600 N were sequentially applied to the occlusal surface of both splinted crowns (Models A and B), as these values reflect typical masticatory forces and parafunctional habit forces associated with excessive biting loads in the posterior mandibular region. The loads were applied vertically and obliquely to simulate chewing forces.

The PDL was modeled as a soft tissue element with a low modulus of elasticity (0.1 MPa) to mimic its role in absorbing and distributing forces during mastication. The base of the model was fixed, simulating the support provided by the alveolar bone.

Simulation scenarios

**Figure 3:**
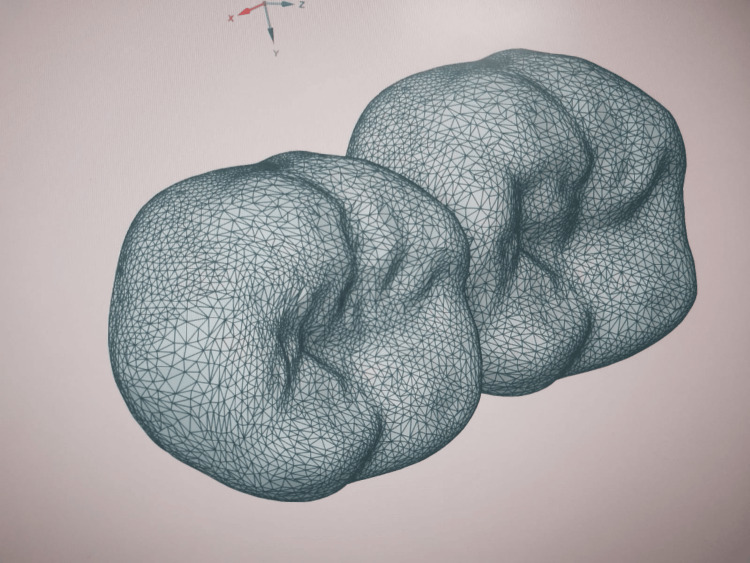
Occlusal view of the nodal structure of the lithium disilicate splinted crown (Model A) Figure was generated by the authors using the SolidWorks software (Dassault Systèmes SolidWorks Corp., Waltham, MA).

**Figure 4:**
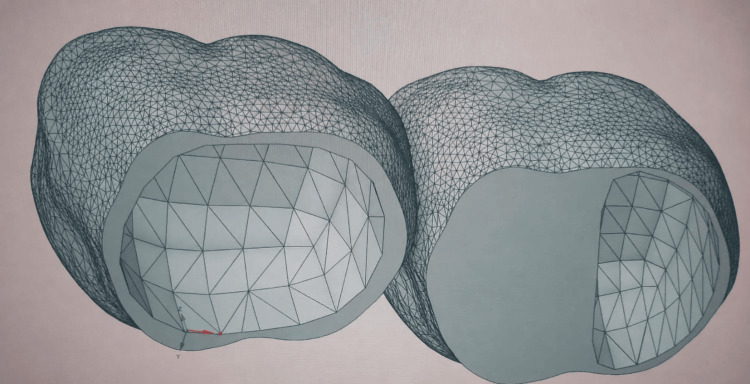
Internal surface view of the nodal structure of the lithium disilicate splinted crown (Model A) Figure was generated by the authors using the SolidWorks software (Dassault Systèmes SolidWorks Corp., Waltham, MA).

**Figure 5:**
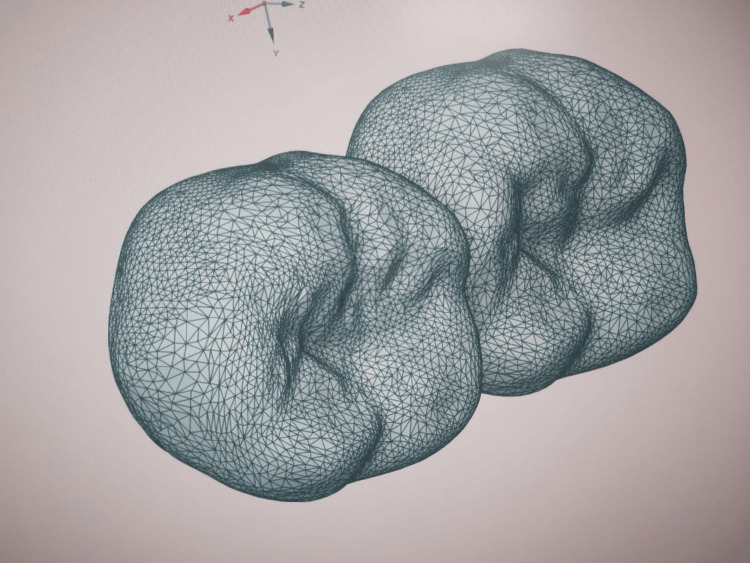
Occlusal view of the nodal structure of the zirconia splinted crown (Model B) Figure was generated by the authors using the SolidWorks software (Dassault Systèmes SolidWorks Corp., Waltham, MA).

**Figure 6:**
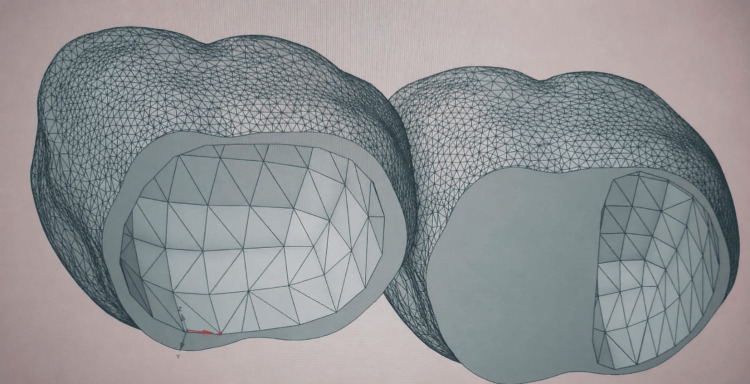
Internal surface view of the nodal structure of the zirconia splinted crown (Model B) Figure was generated by the authors using the SolidWorks software (Dassault Systèmes SolidWorks Corp., Waltham, MA).

Two main models were analyzed to evaluate the effects of different splinting materials on stress distribution in both healthy and hemisected teeth. Model A consisted of a lithium disilicate splinted crown on the mandibular first molar and the hemisected mandibular second molar. Figure 3 and Figure 4 show the occlusal and internal surface views of the nodal structure of the lithium disilicate splinted crown, respectively. Model B consisted of a zirconia splinted crown on the mandibular first molar and the hemisected mandibular second molar. Figure 5 and Figure 6 show the occlusal and internal surface views of the nodal structure of the zirconia splinted crown, respectively.

For each model, simulations were performed under both vertical and oblique loading conditions to evaluate the influence of each material on stress distribution in the remaining tooth structure, including the pulp chamber, dentin-enamel junction, and the PDL.

Stress analysis and post-processing

The von Mises stress criterion was used to evaluate the mechanical performance of each restoration. Von Mises stress represents the equivalent stress generated within a material under applied loading. It is commonly used in FEA to identify areas of stress concentration and assess the likelihood of material failure. Therefore, it was selected as the primary outcome measure in this study. The results were visualized using stress maps, which highlighted regions of high stress concentration in both the tooth structure and the splinted crowns.

**Figure 7:**
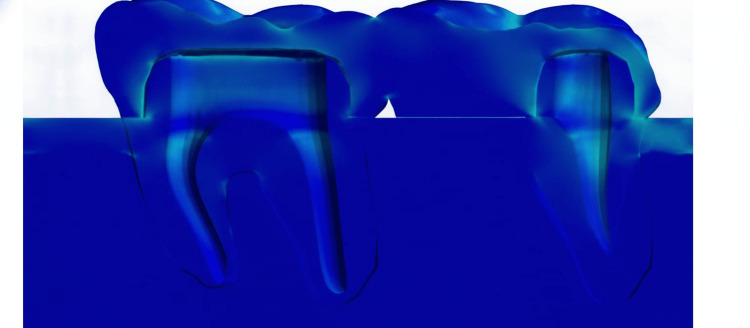
Overview of stress distribution in the finite element model after force loading Figure was generated by the authors using the SolidWorks software (Dassault Systèmes SolidWorks Corp., Waltham, MA).

The stress distribution was compared across the two models (lithium disilicate and zirconia) (Figure 7) to determine which material provided the most favorable distribution of forces, reducing the risk of failure at critical points such as the pulp and PDL interface. Key metrics, such as peak stress values and the locations of maximum stress concentrations, were used for comparison.

## Results

**Figure 8:**
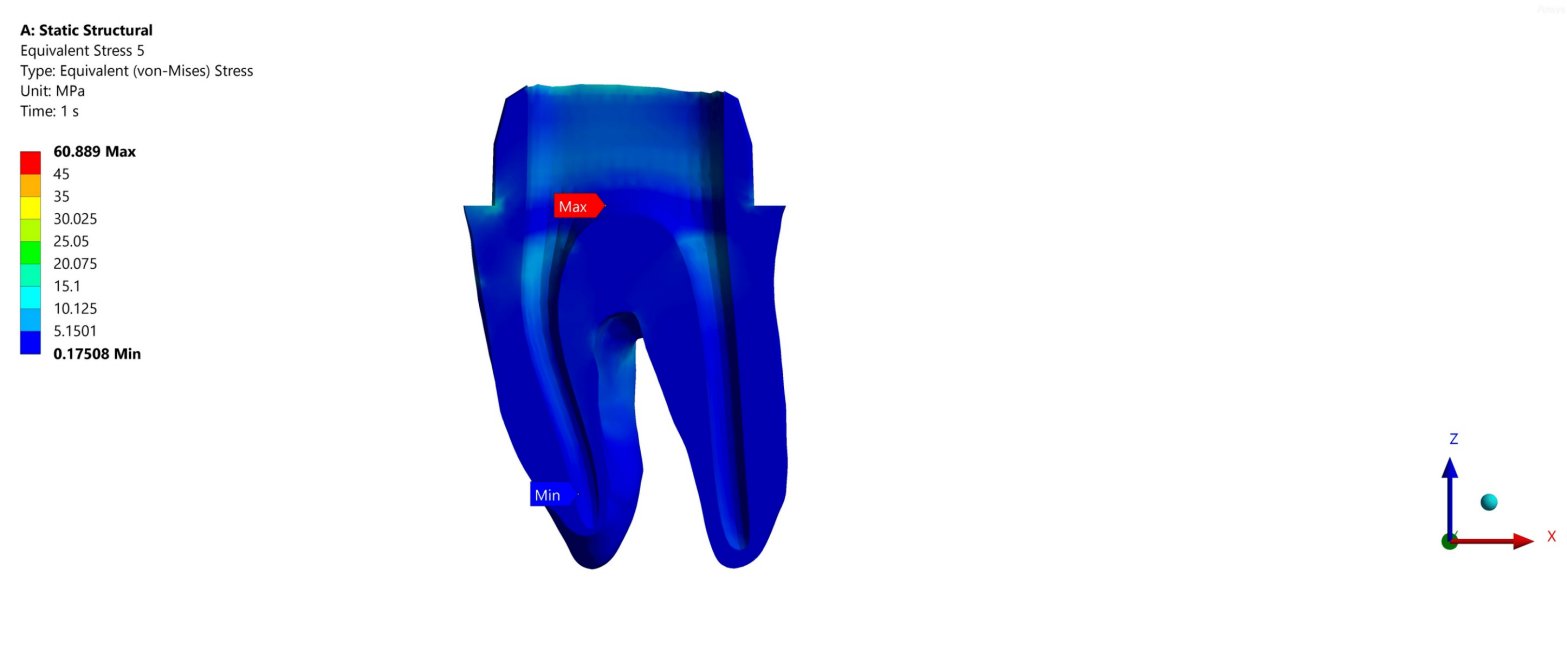
Stress distribution in the adjacent tooth restored with a lithium disilicate splinted crown under a 450 N load Figure was generated by the authors using the SolidWorks software (Dassault Systèmes SolidWorks Corp., Waltham, MA).

**Figure 9:**
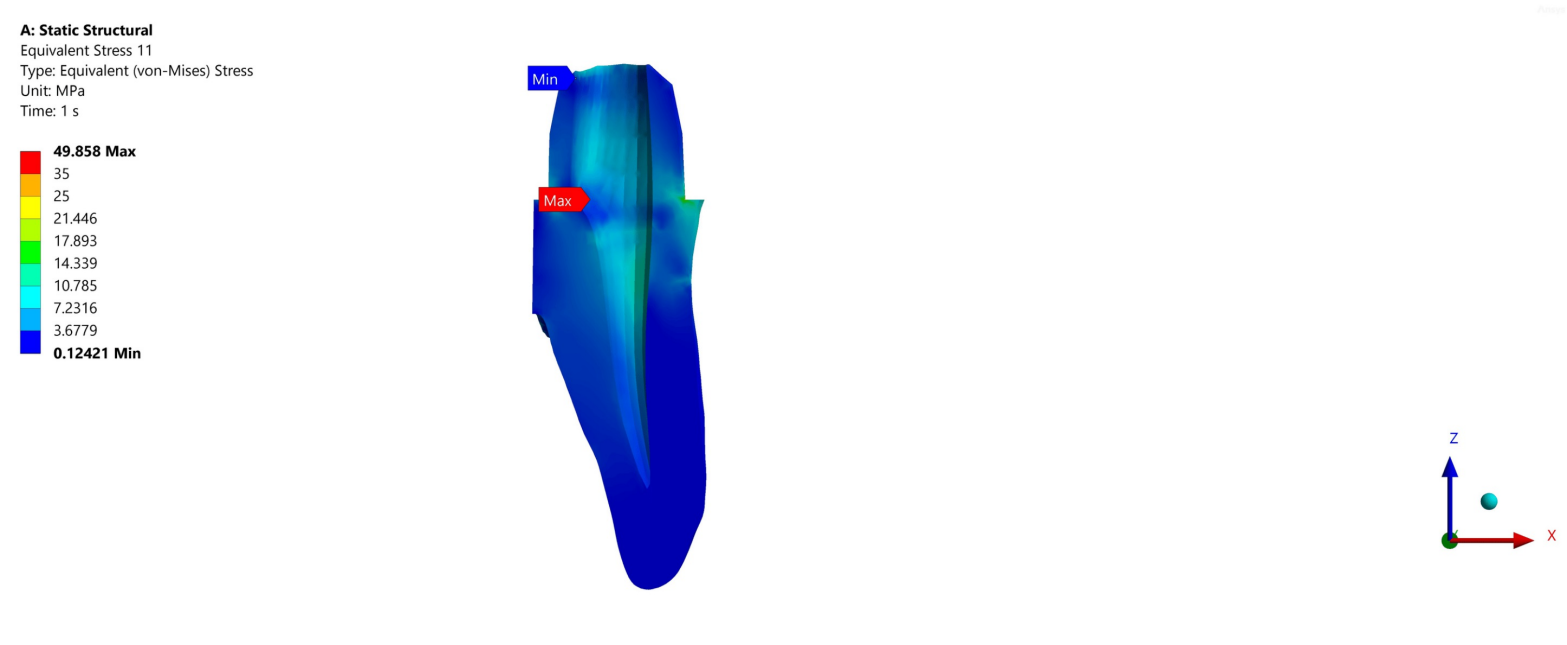
Stress distribution in the hemisected tooth restored with a lithium disilicate splinted crown under a 450 N load Figure was generated by the authors using the SolidWorks software (Dassault Systèmes SolidWorks Corp., Waltham, MA).

**Figure 10:**
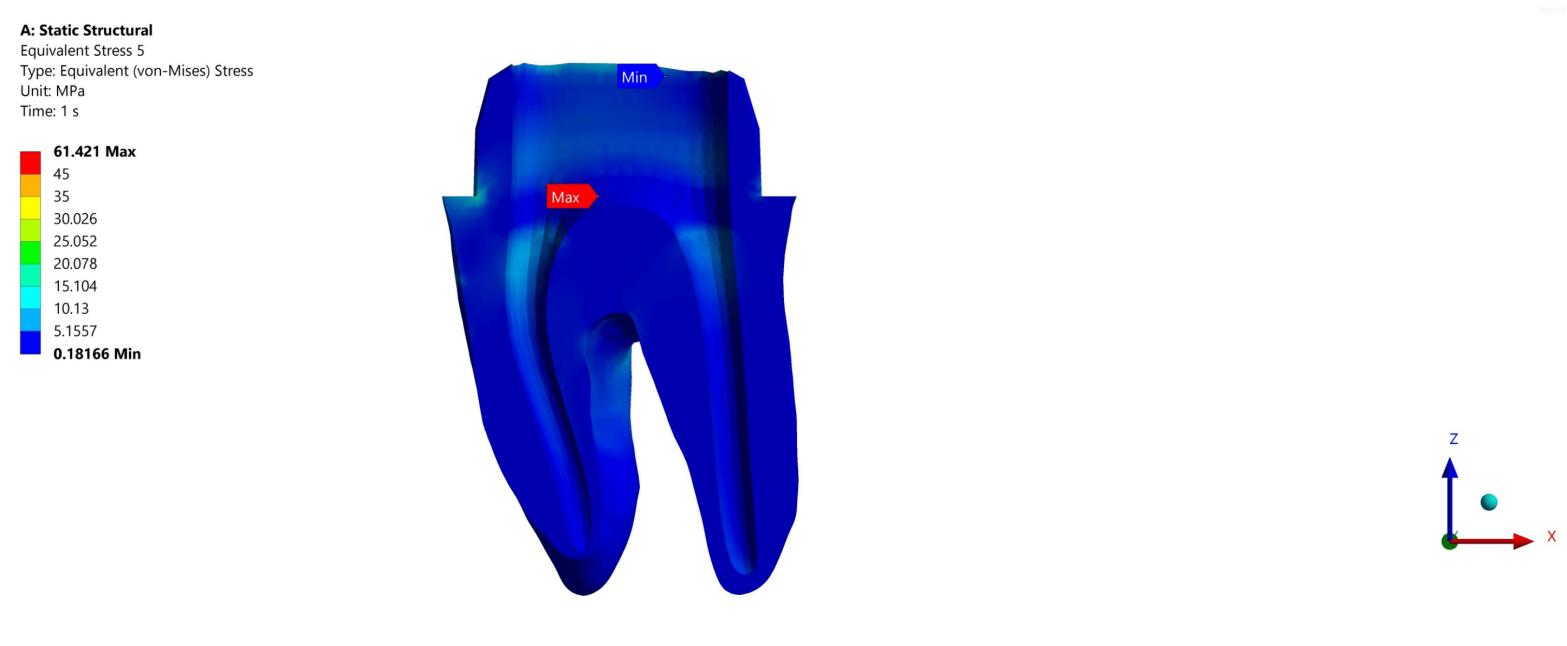
Stress distribution in the adjacent tooth restored with a zirconia splinted crown under a 450 N load Figure was generated by the authors using the SolidWorks software (Dassault Systèmes SolidWorks Corp., Waltham, MA).

**Figure 11:**
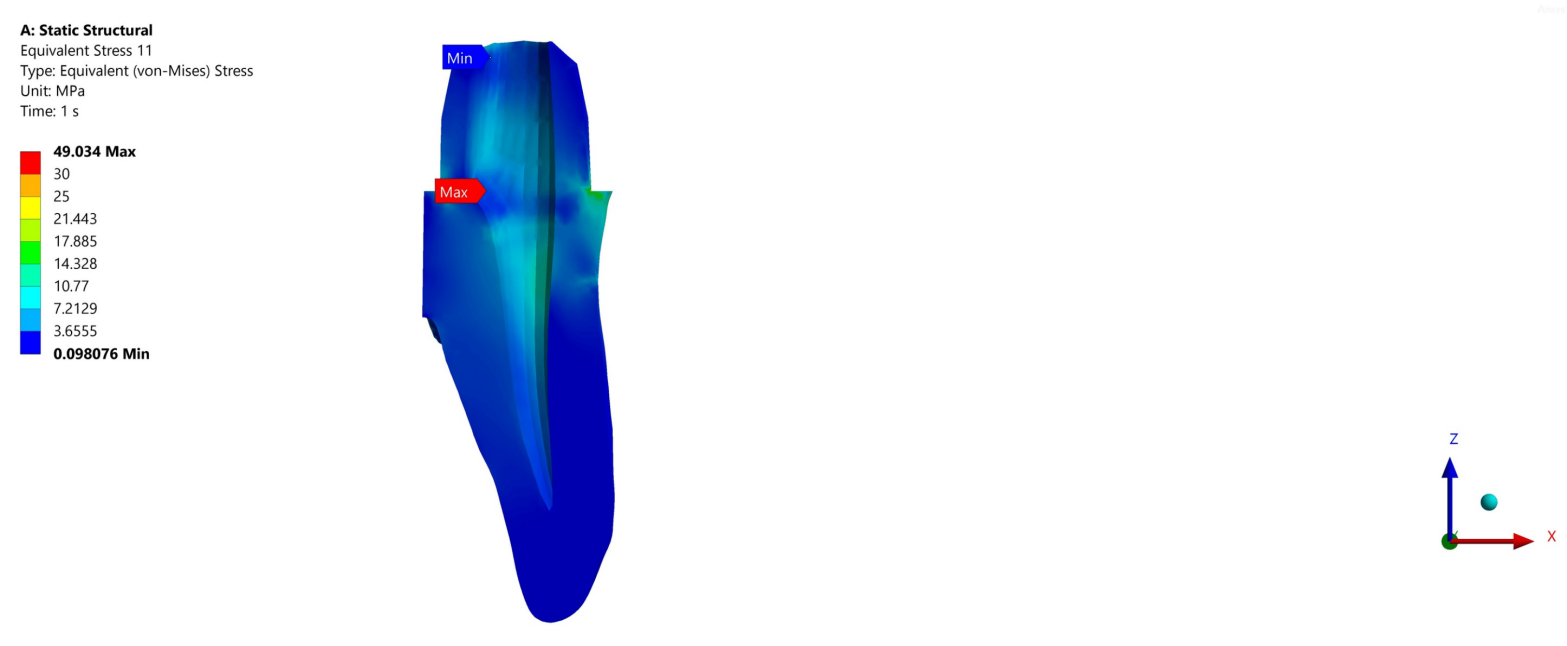
Stress distribution in the hemisected tooth restored with a zirconia splinted crown under a 450 N load Figure was generated by the authors using the SolidWorks software (Dassault Systèmes SolidWorks Corp., Waltham, MA).

The loading forces were applied vertically. Under a 450 N load, the maximum stress for the lithium disilicate splinted crown was 60.889 MPa on the adjacent tooth (Figure 8) and 49.858 MPa on the hemisected tooth (Figure 9), whereas the maximum stress for the zirconia splinted crown was 61.421 MPa on the adjacent tooth (Figure 10) and 49.034 MPa on the hemisected tooth (Figure 11).

**Figure 12:**
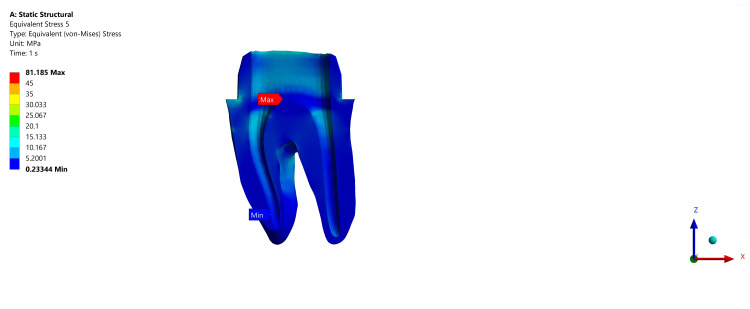
Stress distribution in the adjacent tooth restored with a lithium disilicate splinted crown under a 600 N load Figure was generated by the authors using the SolidWorks software (Dassault Systèmes SolidWorks Corp., Waltham, MA).

**Figure 13:**
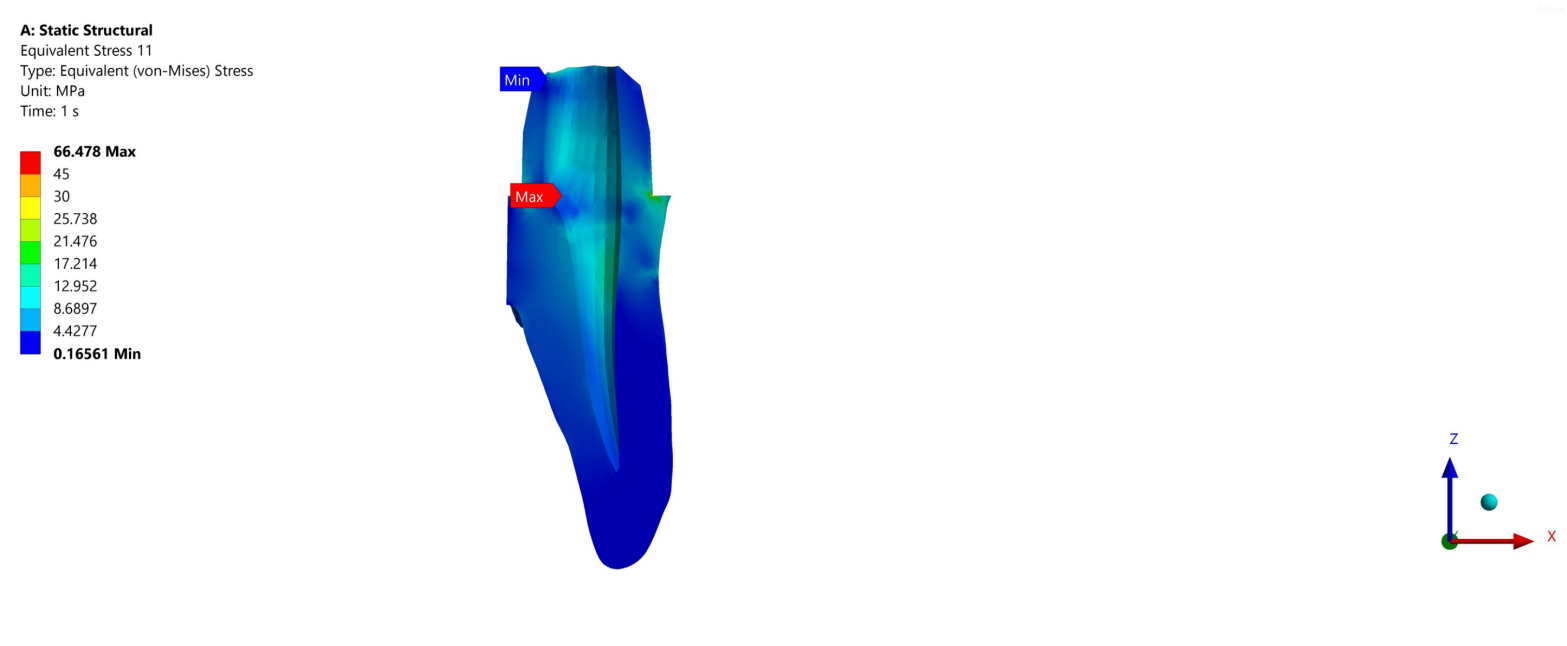
Stress distribution in the hemisected tooth restored with a lithium disilicate splinted crown under a 600 N load Figure was generated by the authors using the SolidWorks software (Dassault Systèmes SolidWorks Corp., Waltham, MA).

**Figure 14:**
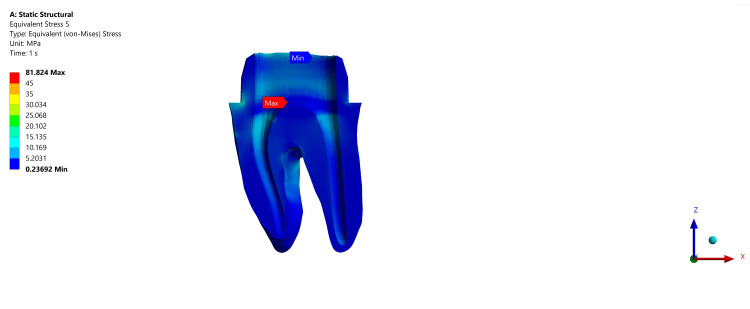
Stress distribution in the adjacent tooth restored with a zirconia splinted crown under a 600 N load Figure was generated by the authors using the SolidWorks software (Dassault Systèmes SolidWorks Corp., Waltham, MA).

**Figure 15:**
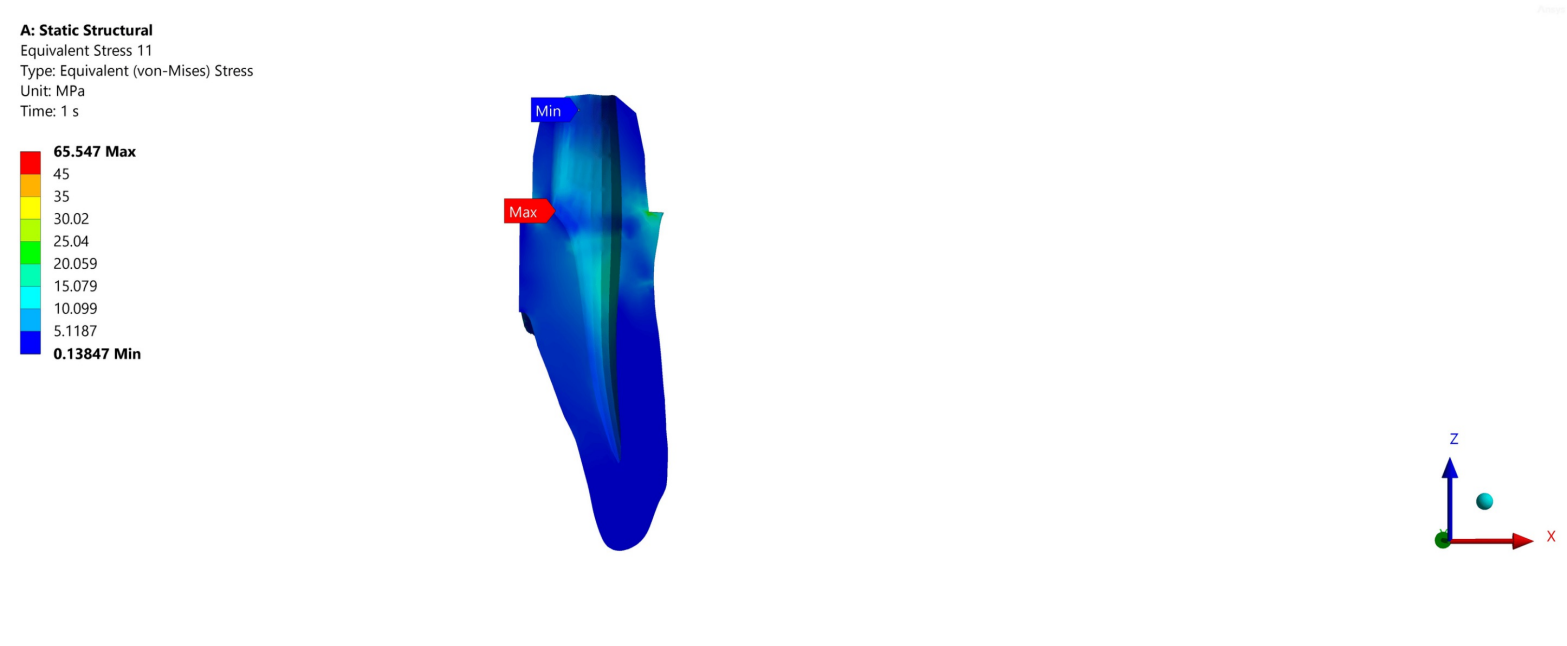
Stress distribution in the hemisected tooth restored with a zirconia splinted crown under a 600 N load Figure was generated by the authors using the SolidWorks software (Dassault Systèmes SolidWorks Corp., Waltham, MA).

Under a 600 N load, the maximum stress for the lithium disilicate splinted crown was 81.185 MPa on the adjacent tooth (Figure 12) and 66.478 MPa on the hemisected tooth (Figure 13), whereas the maximum stress for the zirconia splinted crown was 81.824 MPa on the adjacent tooth (Figure 14) and 65.547 MPa on the hemisected tooth (Figure 15).

A summary of the results is presented in Table 1.

**Table 1:** Maximum von Mises stress values (MPa) in hemisected and adjacent teeth restored with lithium disilicate and zirconia splinted crowns under different loading conditions Maximum von Mises stress values obtained from FEA of hemisected mandibular molars restored with lithium disilicate and zirconia splinted crowns under static loads of 450 N and 600 N. Lower stress values indicate more favorable stress distribution within the remaining tooth structure. FEA, finite element analysis

Load (N)	Lithium disilicate (hemisected tooth)	Zirconia (hemisected tooth)	Lithium disilicate (adjacent tooth)	Zirconia (adjacent tooth)
450	49.858	49.034	60.889	61.421
600	66.478	65.547	81.185	81.824

## Discussion

Hemisection of teeth is often performed in cases of extensive decay, fracture, or periodontal disease, in which part of the tooth is removed while preserving the remaining healthy tooth structure. The use of splinted crowns in such cases is a common restorative approach that helps reduce stress concentrations and improve the prognosis of the remaining tooth. 

Impact of crown material on stress distribution

The selection of crown material plays a pivotal role in the biomechanical performance of dental restorations, particularly concerning stress distribution within the restored tooth and adjacent structures. FEA studies have demonstrated that materials with higher elastic moduli, such as zirconia and alumina-based ceramics, tend to concentrate stress within the crown itself, thereby reducing stress transmission to the underlying tooth structure. For instance, D'Souza and Aras (2017) found that alumina-based all-ceramic crowns exhibited the highest von Mises stresses within the crown but the least stress distribution to the surrounding tooth structure, suggesting a protective effect on the tooth [16].

Conversely, materials with lower stiffness, such as resin-based composites, may absorb occlusal forces more effectively, potentially leading to more uniform stress distribution across the tooth-restoration complex. Ausiello et al. (2023) reported that resin-based computer-aided design/computer-aided manufacturing (CAD/CAM) crowns generated lower tensile and shear stresses at the crown-cement interface, which could enhance the longevity of the restoration [17].

Tuğrul Aslan et al. conducted a study on hemisected teeth restored with single crowns as their coronal restorations, but they suggested that splinted crowns may be preferable to single crowns for hemisected teeth and that further studies could enhance the existing literature [11].

As studies evaluating the stress distribution of splinted crowns on hemisected teeth are lacking, we conducted this study to analyze this property in zirconia and lithium disilicate, as these are among the most commonly used materials for postendodontic restorations.

We found that zirconia splinted crowns transferred less stress to the hemisected tooth than lithium disilicate crowns. This may be attributed to the difference in their flexural strengths, 1000 MPa and 400 MPa, respectively.

The study of stress distribution in splinted crowns on hemisected teeth through FEA plays a significant role in understanding how these restorations behave under functional loading and how they can improve the longevity and stability of compromised teeth. Singh et al. demonstrated through 3D FEA that variations in supporting bone conditions and restorative post designs significantly influence stress distribution patterns in endodontically treated teeth, emphasizing the importance of biomechanical considerations during restorative treatment planning [18].

Clinical implications and recommendations

The results of FEA studies have significant clinical implications for restorative dentistry, particularly for the management of hemisected teeth. By evaluating stress distribution, FEA can guide clinicians in choosing the most appropriate materials and techniques for restoring compromised teeth. The use of splinted crowns, especially those made from zirconia or other high-strength materials, can improve the prognosis of hemisected teeth by preventing excessive stress accumulation and reducing the likelihood of fractures.

Clinicians should consider the biomechanical properties of both the tooth structure and the restoration material when planning treatments for hemisected teeth. A 3D FEA by Kumar et al. demonstrated that the type and diameter of post systems significantly alter stress concentration patterns within endodontically treated teeth, highlighting the importance of restorative material selection in minimizing biomechanical failure [19]. Additionally, FEA can be used as a valuable tool in treatment planning and decision-making, allowing for a more tailored approach to each patient's needs.

Future research directions

While FEA has provided valuable insights into the stress distribution of splinted crowns on hemisected teeth, there are still areas that require further investigation. Future studies could explore the long-term effects of different splinting techniques and materials on the longevity and success of hemisected teeth. Additionally, research could focus on the influence of varying occlusal loads, such as those associated with bruxism, on the performance of splinted crowns.

Future studies could also investigate the use of nanoceramics and their stress distribution in splinted crowns.

## Conclusions

From a clinical perspective, reduced stress concentration within the remaining tooth structure may decrease the risk of root fracture, restoration failure, and biomechanical complications in hemisected teeth. The higher flexural strength and elastic modulus of zirconia may contribute to its superior ability to withstand masticatory forces and transfer loads more evenly across the splinted unit. Consequently, zirconia splinted crowns may be considered a preferable restorative option for hemisected molars subjected to high occlusal loads, particularly in patients with parafunctional habits or heavy masticatory function. Within the limitations of this study, we conclude that zirconia splinted crowns exhibited lower von Mises stress in the hemisected tooth than lithium disilicate splinted crowns, indicating more favorable stress distribution under the simulated loading conditions. The use of splinted crowns in hemisected teeth significantly improves stress distribution, helping to prevent fractures and enhance the overall stability of the tooth. However, although numerical differences were observed between the materials, these differences were not statistically significant. Therefore, no definitive superiority of one material over the other can be established based on the present study. FEA has proven to be a highly effective tool for simulating and analyzing stress distribution in dental restorations, providing valuable insights into the impact of splinting techniques and materials. As restorative dentistry continues to evolve, FEA will remain an essential tool in optimizing treatment strategies for compromised teeth, ensuring better clinical outcomes for patients.
